# Supplementary figures and images for: Ecosystem engineers drive differing microbial community composition in intertidal estuarine sediments
Source: PLoS One. 2021 Feb 19;16(2):e0240952. doi: 10.1371/journal.pone.0240952 (PMC7895378; doi:10.1371/journal.pone.0240952)

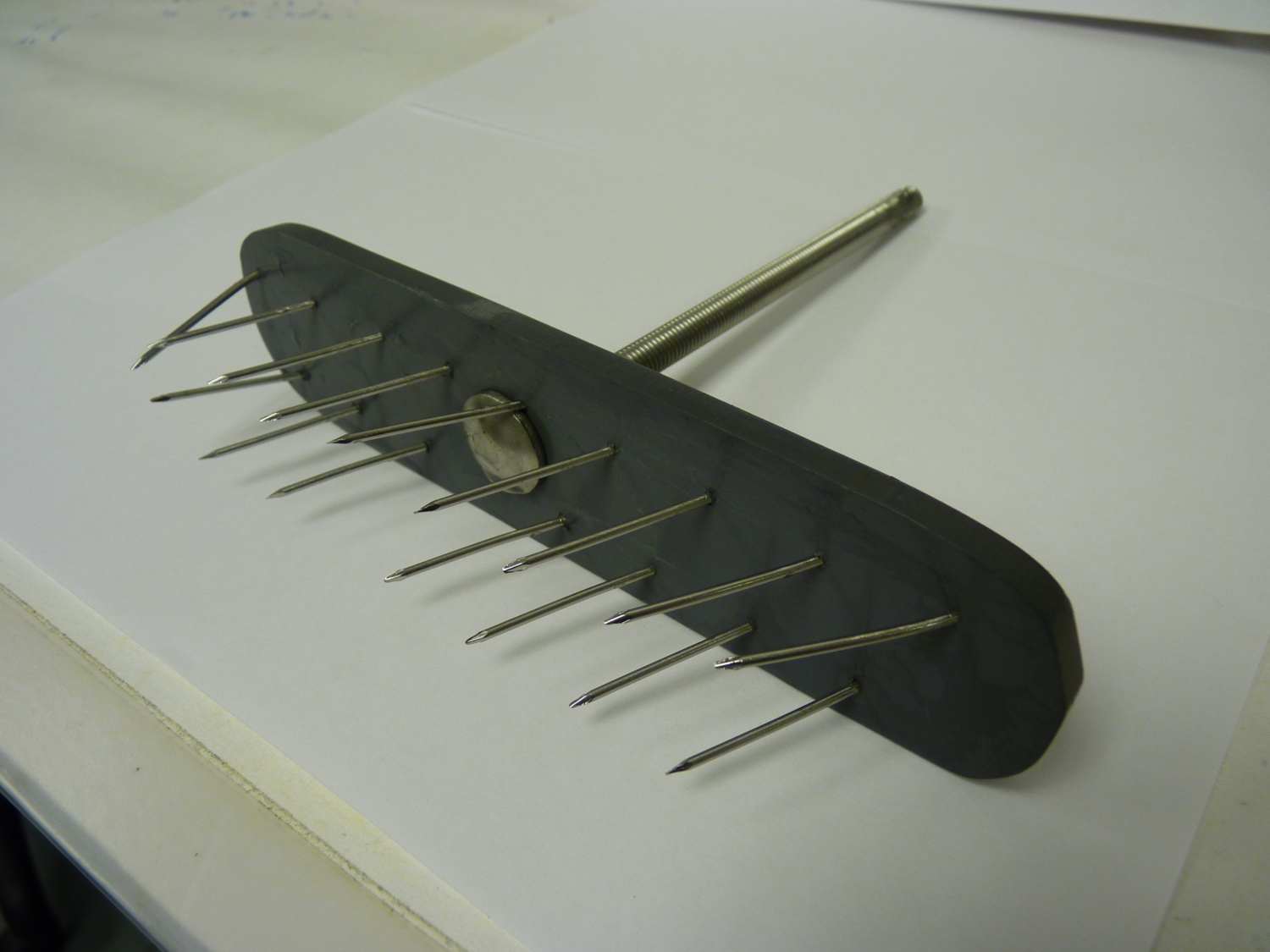

Supplement: S1 Fig — It consists of an acrylic base (40 mm width, 180 mm length, 6 mm depth) fixed to the head of an M8 200 mm A2 stainless steel square neck carriage bolt using an A2 stainless steel wing nut, with a threaded plastic T-handle. Eighteen stainless steel panel pins (1.5 mm x 40 mm) are inserted through two rows of 1 mm width holes drilled at a 65° angle from the base, leaving the pins extruding roughly 30 mm below the bottom surface of the base. Rows were 20 mm apart, with pins within each row 20 mm apart. Rows were offset by 10 mm, resulting in a pin disturbing the sediment every 10 mm at 65° and 115° to a depth of 30 mm when turned through a full circle. (TIF) [file pone.0240952.s001.tif]

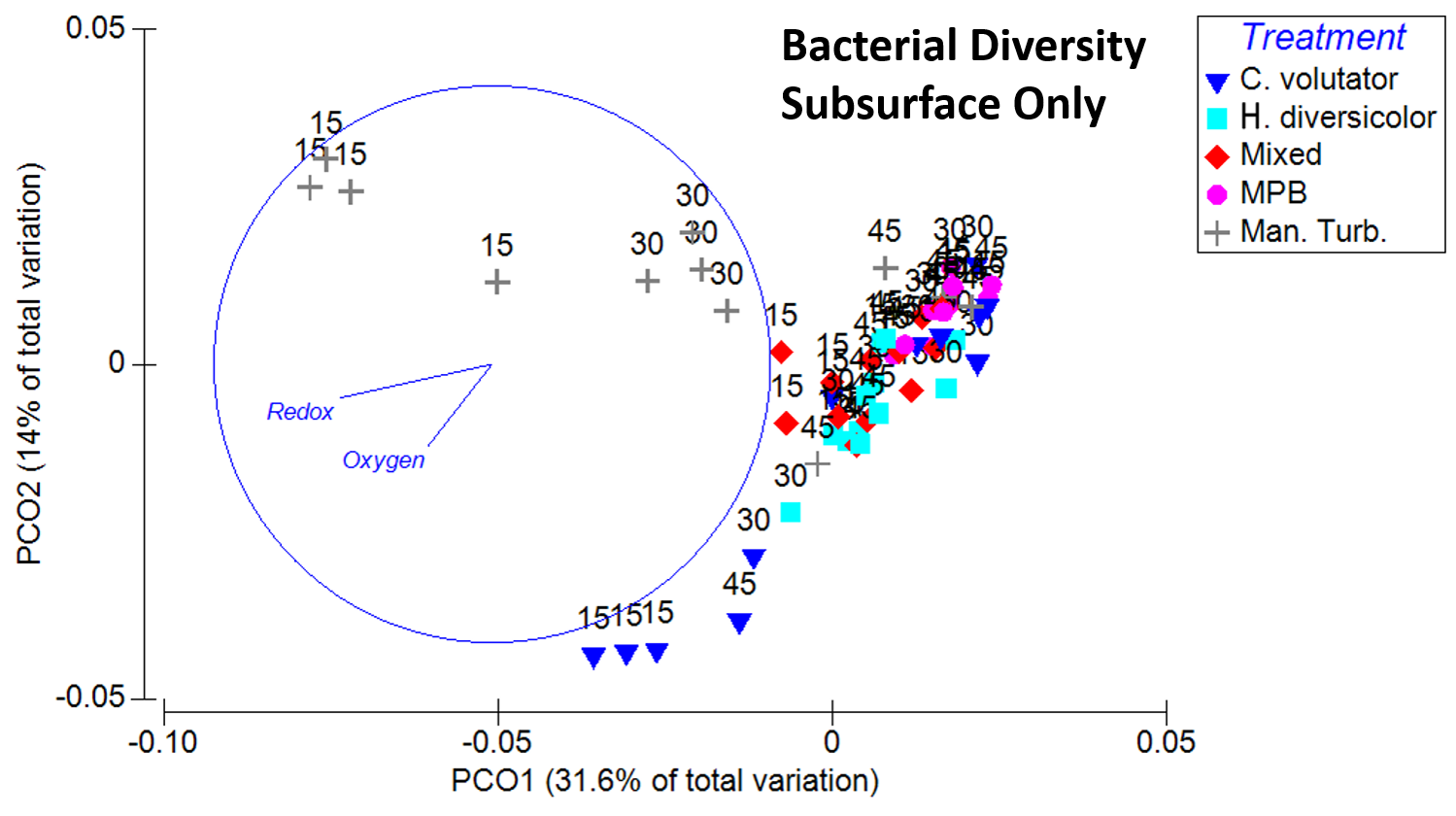

Supplement: S2 Fig — Ordination after standardisation of ASVs and a weighted Unifrac distance matrix. Redox potential and dissolved oxygen are overlaid as vectors. C. v.–C. volutator; H. d.–H. diversicolor; Mixed–Mixed infauna; MPB–Microphytobenthos only; Man. Turb.–Manual-turbation. (TIF) [file pone.0240952.s002.tif]
