# Supplementary material for: Ecosystem engineers drive differing microbial community composition in intertidal estuarine sediments
Source: PLoS One. 2021 Feb 19;16(2):e0240952. doi: 10.1371/journal.pone.0240952 (PMC7895378; doi:10.1371/journal.pone.0240952)
Supplement: S1 Table — (DOCX) [file pone.0240952.s003.docx]

S1 Table. Relative abundance of diatom species identified in surface sediments.

| **Species** | ***C. volutator*** | ***H. diversicolor*** | **Mixed** | **MPB** | **Manual Turbation** |
| --- | --- | --- | --- | --- | --- |
| *Achnanthes conspica* | 0.64 | 0.72 | 0.48 | 0.40 | 0.25 |
| *Achnanthes delicatula* | 6.02 | 5.03 | 4.34 | 3.01 | 7.38 |
| *Achnanthes hauckiana* | 12.34 | 9.29 | 7.01 | 7.56 | 11.57 |
| *Achnanthes lanceolata* | 7.09 | 5.28 | 6.44 | 5.13 | 11.91 |
| *Achnanthes lanceolata - V* | 4.34 | 3.03 | 3.78 | 4.28 | 8.34 |
| *Amphipora angustata* | 0.24 | 0.24 | 0.32 | 0.89 | 0.00 |
| *Amphora acutiuscula* | 1.19 | 0.87 | 1.04 | 1.47 | 0.83 |
| *Amphora hylina* | 0.25 | 0.49 | 0.16 | 0.41 | 0.25 |
| *Amphora ostrearia* | 2.42 | 1.52 | 0.96 | 1.13 | 1.56 |
| *Amphora sp. 1* | 0.48 | 0.08 | 0.56 | 1.30 | 0.08 |
| *Amphora sp. 2* | 0.24 | 0.08 | 0.48 | 0.16 | 0.33 |
| *Cocconeis disculus* | 2.65 | 1.92 | 1.21 | 0.97 | 2.39 |
| *Cocconeis placentula* | 0.08 | 1.27 | 0.00 | 0.08 | 0.08 |
| *Cocconeis speciosa* | 0.32 | 0.24 | 0.08 | 0.08 | 0.50 |
| *Cocconeis sp. 1* | 5.92 | 4.71 | 5.72 | 3.14 | 6.77 |
| *Fragilaria schulzi* | 0.80 | 1.67 | 1.12 | 0.73 | 1.32 |
| *Fragilaria sp. 3* | 0.16 | 0.16 | 0.16 | 0.40 | 0.33 |
| *Fragilaria sp. 4* | 0.16 | 0.24 | 0.08 | 0.64 | 0.91 |
| *Gyrosigma wansbeckii* | 0.00 | 0.16 | 0.16 | 0.40 | 0.08 |
| *Navicula arenaria* | 0.08 | 0.32 | 0.00 | 0.00 | 0.89 |
| *Navicula cincta* | 3.55 | 2.32 | 4.28 | 2.11 | 4.54 |
| *Navicula clementis* | 4.11 | 4.97 | 4.27 | 2.94 | 5.24 |
| *Navicula elegans* | 0.48 | 0.32 | 0.24 | 0.33 | 0.66 |
| *Navicula exigua* | 0.80 | 0.40 | 0.89 | 0.97 | 1.90 |
| *Navicula maculosa* | 0.48 | 0.88 | 0.48 | 0.48 | 1.24 |
| *Navicula pelliculosa* | 1.12 | 0.48 | 0.72 | 0.24 | 1.64 |
| *Navicula ramosissima* | 2.33 | 1.91 | 2.97 | 1.14 | 0.41 |
| *Navicula rotaeana* | 0.58 | 0.65 | 1.38 | 0.73 | 0.81 |
| *Navicula salinarum* | 1.44 | 7.13 | 2.57 | 1.96 | 2.07 |
| *Navicula sp. 1* | 1.28 | 1.04 | 2.08 | 1.45 | 3.39 |
| *Navicula sp. 2* | 2.26 | 0.48 | 1.62 | 1.28 | 2.54 |
| *Navicula sp. 4* | 0.08 | 0.00 | 1.04 | 0.00 | 0.08 |
| *Navicula sp. 7* | 0.80 | 0.64 | 1.92 | 1.61 | 1.33 |
| *Nitzschia behrei* | 0.00 | 0.00 | 0.00 | 0.00 | 0.91 |
| *Nitzschia biblobata* | 0.32 | 0.57 | 0.16 | 0.48 | 0.33 |
| *Nitzschia closterium* | 8.53 | 1.36 | 2.99 | 4.73 | 0.75 |
| *Nitzschia dissipata* | 0.72 | 0.41 | 0.97 | 1.38 | 0.83 |
| *Nitzschia dubia* | 0.00 | 0.00 | 0.32 | 0.00 | 0.58 |
| *Nitzschia laevis* | 11.48 | 29.77 | 26.75 | 32.09 | 0.83 |
| *Nitzschia hungarica* | 0.00 | 0.00 | 0.24 | 0.00 | 0.00 |
| *Nitzschia kutzingina* | 1.20 | 0.48 | 0.48 | 1.53 | 0.82 |
| *Nitzschia sp. 1* | 1.04 | 0.72 | 1.53 | 2.42 | 0.82 |
| *Nitzschia sp. 2* | 0.40 | 0.56 | 0.40 | 0.73 | 0.42 |
| *Nitzschia sp. 4* | 0.90 | 0.64 | 0.97 | 0.96 | 0.91 |
| *Nitzschia sp. 14* | 0.16 | 0.00 | 0.00 | 0.40 | 0.00 |
| *Opephora martyi* | 1.28 | 1.13 | 0.88 | 1.15 | 1.81 |
| *Opephora schwartzii* | 3.31 | 1.91 | 2.57 | 2.78 | 2.95 |
| *Opephora sp. 1* | 0.81 | 0.16 | 0.40 | 0.24 | 0.89 |
| *Pleurosigma marinum* | 0.00 | 0.00 | 0.00 | 0.33 | 0.00 |
| *Rhaphoneis amphiceros* | 0.24 | 0.16 | 0.16 | 0.00 | 0.00 |
| *Rhaphoneis surirella* | 4.33 | 3.13 | 2.25 | 2.86 | 4.93 |
| *Surirella sp. 2* | 0.58 | 0.08 | 0.32 | 0.49 | 0.57 |
| *Unknown* 1 | 0.00 | 0.40 | 0.00 | 0.00 | 0.00 |
