# Supplementary material for: Ecosystem engineers drive differing microbial community composition in intertidal estuarine sediments
Source: PLoS One. 2021 Feb 19;16(2):e0240952. doi: 10.1371/journal.pone.0240952 (PMC7895378; doi:10.1371/journal.pone.0240952)
Supplement: S2 Table — Species included up to a cumulative contribution to dissimilarity of 40%. C. v.–C. volutator; H. d.–H. diversicolor; Mixed–Mixed infauna; MPB–Microphytobenthos only; Man. Turb.–Manual-turbation. Rel. abun.–relative abundance; mean abun. diff.- mean abundance difference; diss.- dissimilarity. (DOCX) [file pone.0240952.s004.docx]

S2 Table. SIMPER analysis of pairwise treatments identified as significantly different by ANOSIM. Species included up to a cumulative contribution to dissimilarity of 40%. *C. v.* – *C. volutator; H. d. – H. diversicolor*; Mixed – Mixed infauna; MPB – Microphytobenthos only; Man. Turb. – Manual-turbation. Rel. abun. – relative abundance; mean abun. diff.- mean abundance difference; diss.- dissimilarity.

| **Treatment 1 (T_1_)** | **Treatment 2 (T_2_)** | **Pairwise diss.** | **Species** | **T_1_ rel. abun.** | **T_2_ rel. abun.** | **Mean abun. diff.** | **Cumulative contribution to diss. (%)** |
| --- | --- | --- | --- | --- | --- | --- | --- |
| ***C. v.*** | ***H. d.*** | 39.93 | *Nitzschia laevis* | 11.48 | 29.77 | 9.15 | 22.91 |
|  |  |  | *Nitzschia closterium* | 8.53 | 1.36 | 3.59 | 31.89 |
|  |  |  | *Navicula salinarum* | 1.44 | 7.13 | 2.84 | 39.01 |
|  |  |  | *Achnanthes hauckiana* | 12.34 | 9.29 | 1.87 | 43.69 |
| ***C. v.*** | **Man. Turb.** | 39.89 | *Nitzschia laevis* | 11.48 | 0.83 | 5.33 | 13.35 |
|  |  |  | *Nitzschia closterium* | 8.53 | 0.75 | 3.89 | 23.10 |
|  |  |  | *Achnanthes lanceolata* | 7.09 | 11.91 | 2.45 | 29.24 |
|  |  |  | *Achnanthes hauckiana* | 12.34 | 11.57 | 2.32 | 35.06 |
|  |  |  | *Achnanthes lanceolata* V | 4.34 | 8.34 | 2.22 | 40.63 |
| ***H. d.*** | **Man. Turb.** | 50.06 | *Nitzschia laevis* | 29.77 | 0.83 | 14.47 | 28.91 |
|  |  |  | *Achnanthes lanceolata* | 5.28 | 11.91 | 3.32 | 35.53 |
|  |  |  | *Achnanthes lanceolata* V | 3.03 | 8.34 | 2.81 | 41.14 |
| **Mixed** | **Man. Turb.** | 47.97 | *Nitzschia laevis* | 26.75 | 0.83 | 12.96 | 27.02 |
|  |  |  | *Achnanthes lanceolata* | 6.44 | 11.91 | 2.74 | 32.73 |
|  |  |  | *Achnanthes hauckiana* | 7.01 | 11.57 | 2.57 | 38.08 |
|  |  |  | *Achnanthes lanceolata* V | 3.78 | 8.34 | 2.36 | 42.99 |
| **MPB** | **Man. Turb.** | 52.90 | *Nitzschia laevis* | 32.09 | 0.83 | 15.63 | 29.55 |
|  |  |  | *Achnanthes lanceolata* | 5.13 | 11.91 | 3.39 | 35.96 |
|  |  |  | *Achnanthes hauckiana* | 7.56 | 11.57 | 2.51 | 40.70 |
