# Supplementary material for: Ecosystem engineers drive differing microbial community composition in intertidal estuarine sediments
Source: PLoS One. 2021 Feb 19;16(2):e0240952. doi: 10.1371/journal.pone.0240952 (PMC7895378; doi:10.1371/journal.pone.0240952)
Supplement: S3 Table — Sample statistic (global R): 0.47, p = 0.001. C. v.–C. volutator; H. d.–H. diversicolor; Mixed- Mixed infauna; MPB- Microphytobenthos only; Man. Turb.- Manual turbation. (DOCX) [file pone.0240952.s005.docx]

S3 Table. ANOSIM summary table for diatom assemblage composition between treatment groups. Sample statistic (global R): 0.47, *p* = 0.001. *C. v.* – *C. volutator; H. d. – H. diversicolor*; Mixed- Mixed infauna; MPB- Microphytobenthos only; Man. Turb.- Manual turbation.

| **Groups** | **R statistic** | **Significance level %** | **Possible permutations** | **Actual permutations** | **Number >= observed** |
| --- | --- | --- | --- | --- | --- |
| ***C. v*., *H. d*.** | 0.70 | 2.9 | 35 | 35 | 1 |
| ***C. v*., Mixed** | 0.45 | 5.7 | 35 | 35 | 2 |
| ***C. v*., MPB** | 0.63 | 5.7 | 35 | 35 | 2 |
| ***C. v*., Man. Turb.** | 0.43 | 2.9 | 35 | 35 | 1 |
| ***H. d*., Mixed** | -0.02 | 48.6 | 35 | 35 | 17 |
| ***H. d*., MPB** | 0.26 | 17.1 | 35 | 35 | 6 |
| ***H. d.*, Man. Turb.** | 1.00 | 2.9 | 35 | 35 | 1 |
| **Mixed, MPB** | -0.17 | 80.0 | 35 | 35 | 28 |
| **Mixed, Man. Turb.** | 0.96 | 2.9 | 35 | 35 | 1 |
| **MPB, Man. Turb.** | 0.92 | 2.9 | 35 | 35 | 1 |
