# Supplementary material for: Ecosystem engineers drive differing microbial community composition in intertidal estuarine sediments
Source: PLoS One. 2021 Feb 19;16(2):e0240952. doi: 10.1371/journal.pone.0240952 (PMC7895378; doi:10.1371/journal.pone.0240952)
Supplement: S4 Table — Sequences rarefied at 35000 reads and metrics summarised using treatment medians. C. v.–C. volutator; H. d.–H. diversicolor; Mixed–Mixed infauna; MPB–Microphytobenthos only; Man. Turb.–Manual-turbation. Metrics for source sediment included as T0. (DOCX) [file pone.0240952.s006.docx]

S4 Table. Alpha diversity metrics for bacterial community assemblages. Sequences rarefied at 35000 reads and metrics summarised using treatment medians. *C. v.* – *C. volutator; H. d. – H. diversicolor*; Mixed – Mixed infauna; MPB – Microphytobenthos only; Man. Turb. – Manual-turbation. Metrics for source sediment included as T0.

| **Treatment** | **Depth (mm)** | **ASV richness** | **Shannon's diversity (log e)** | **Pielou's evenness** | **Faith's phylogenetic diversity** |
| --- | --- | --- | --- | --- | --- |
| ***C. v.*** | 0 | 1766 | 6.56 | 0.88 | 113.38 |
| ***H. d.*** | 0 | 1668 | 6.52 | 0.88 | 111.94 |
| **Mixed** | 0 | 1965 | 6.67 | 0.88 | 126.89 |
| **MPB** | 0 | 1582 | 6.43 | 0.87 | 109.26 |
| **Man. Turb.** | 0 | 1656 | 6.32 | 0.86 | 109.20 |
| ***C. v.*** | 15 | 1998 | 6.86 | 0.90 | 127.40 |
| ***H. d.*** | 15 | 1908 | 6.83 | 0.90 | 120.40 |
| **Mixed** | 15 | 1970 | 6.84 | 0.90 | 128.98 |
| **MPB** | 15 | 1728 | 6.80 | 0.91 | 118.12 |
| **Man. Turb.** | 15 | 1779 | 6.55 | 0.88 | 114.10 |
| ***C. v.*** | 30 | 2023 | 6.92 | 0.91 | 129.66 |
| ***H. d.*** | 30 | 1982 | 6.87 | 0.91 | 126.68 |
| **Mixed** | 30 | 1975 | 6.88 | 0.91 | 126.77 |
| **MPB** | 30 | 1900 | 6.86 | 0.91 | 123.19 |
| **Man. Turb.** | 30 | 1699 | 6.75 | 0.91 | 112.55 |
| ***C. v.*** | 45 | 1786 | 6.79 | 0.91 | 119.33 |
| ***H. d.*** | 45 | 1981 | 6.88 | 0.91 | 122.80 |
| **Mixed** | 45 | 1765 | 6.78 | 0.91 | 119.22 |
| **MPB** | 45 | 1753 | 6.79 | 0.91 | 117.96 |
| **Man. Turb.** | 45 | 1750 | 6.80 | 0.91 | 119.23 |
| **T0** | n/a | 1324 | 6.59 | 0.92 | 99.66 |
