# Supplementary material for: Ecosystem engineers drive differing microbial community composition in intertidal estuarine sediments
Source: PLoS One. 2021 Feb 19;16(2):e0240952. doi: 10.1371/journal.pone.0240952 (PMC7895378; doi:10.1371/journal.pone.0240952)
Supplement: S5 Table — Sample statistic (global R): 0.51, p = 0.001. C. v.–C. volutator; H. d.–H. diversicolor; Mixed- Mixed infauna; MPB- Microphytobenthos only; Man. Turb.- Manual turbation. (DOCX) [file pone.0240952.s007.docx]

S5 Table. ANOSIM summary table for bacterial assemblage composition between all treatment groups across all depths. Sample statistic (global R): 0.51, *p* = 0.001. *C. v.* – *C. volutator; H. d. – H. diversicolor*; Mixed- Mixed infauna; MPB- Microphytobenthos only; Man. Turb.- Manual turbation.

| **Groups** | **R Statistic** | **Significance Level %** | **Possible Permutations** | **Actual Permutations** | **Number >= observed** |
| --- | --- | --- | --- | --- | --- |
|  |  |  |  |  |  |
| ***C. v.*, *H. d.*** | 0.45 | 0.1 | 1500625 | 999 | 0 |
| ***C. v*., Mixed** | 0.37 | 0.4 | 1500625 | 999 | 3 |
| ***C. v*., MPB** | 0.42 | 0.1 | 1500625 | 999 | 0 |
| ***C. v.*, Man. Turb.** | 0.66 | 0.1 | 1500625 | 999 | 0 |
| ***H. d*., Mixed** | 0.07 | 18.6 | 1500625 | 999 | 185 |
| ***H. d*., MPB** | 0.65 | 0.1 | 1500625 | 999 | 0 |
| ***H. d*., Man. Turb.** | 0.77 | 0.1 | 1500625 | 999 | 0 |
| **Mixed, MPB** | 0.44 | 0.1 | 1500625 | 999 | 0 |
| **Mixed, Man. Turb.** | 0.75 | 0.1 | 1500625 | 999 | 0 |
| **MPB, Man. Turb.** | 0.71 | 0.1 | 1500625 | 999 | 0 |
