# Supplementary material for: Ecosystem engineers drive differing microbial community composition in intertidal estuarine sediments
Source: PLoS One. 2021 Feb 19;16(2):e0240952. doi: 10.1371/journal.pone.0240952 (PMC7895378; doi:10.1371/journal.pone.0240952)
Supplement: S8 Table — Sample statistic (global R): 0.28, p = 0.001. C. v.–C. volutator; H. d.–H. diversicolor; Mixed- Mixed infauna; MPB- Microphytobenthos only; Man. Turb.- Manual turbation. (DOCX) [file pone.0240952.s010.docx]

S8 Table. ANOSIM summary table for bacterial assemblage composition between all treatment groups across all depths for subsurface sediments only. Sample statistic (global R): 0.28, *p* = 0.001. *C. v.* – *C. volutator; H. d. – H. diversicolor*; Mixed- Mixed infauna; MPB- Microphytobenthos only; Man. Turb.- Manual turbation.

| **Groups** | **R Statistic** | **Significance Level %** | **Possible Permutations** | **Actual Permutations** | **Number >= observed** |
| --- | --- | --- | --- | --- | --- |
|  |  |  |  |  |  |
| **15, 30** | 0.35 | 0.1 | 52521875 | 999 | 0 |
| **15, 45** | 0.40 | 0.3 | 52521875 | 999 | 2 |
| **30, 45** | 0.11 | 9.2 | 52521875 | 999 | 91 |
