# Supplementary material for: Ecosystem engineers drive differing microbial community composition in intertidal estuarine sediments
Source: PLoS One. 2021 Feb 19;16(2):e0240952. doi: 10.1371/journal.pone.0240952 (PMC7895378; doi:10.1371/journal.pone.0240952)
Supplement: S9 Table — Sample statistic (global R): 0.39, p = 0.001. C. v.–C. volutator; H. d.–H. diversicolor; Mixed- Mixed infauna; MPB- Microphytobenthos only; Man. Turb.- Manual turbation. (DOCX) [file pone.0240952.s011.docx]

S9 Table. ANOSIM summary table for bacterial assemblage composition between all depths across all treatment groups for subsurface sediments only. Sample statistic (global R): 0.39, *p* = 0.001. *C. v.* – *C. volutator; H. d. – H. diversicolor*; Mixed- Mixed infauna; MPB- Microphytobenthos only; Man. Turb.- Manual turbation.

| **Groups** | **R Statistic** | **Significance Level %** | **Possible Permutations** | **Actual Permutations** | **Number >= observed** |
| --- | --- | --- | --- | --- | --- |
|  |  |  |  |  |  |
| ***C. v.*, Man. Turb.** | 0.54 | 0.5 | 42875 | 999 | 4 |
| ***C. v.*, Mixed** | 0.22 | 3.6 | 42875 | 999 | 35 |
| ***C. v.*, MPB** | 0.27 | 3.8 | 42875 | 999 | 37 |
| ***C. v.*, *H. d.*** | 0.26 | 1.3 | 42875 | 999 | 12 |
| **Man. Turb., Mixed** | 0.69 | 0.1 | 42875 | 999 | 0 |
| **Man. Turb., MPB** | 0.60 | 0.2 | 42875 | 999 | 1 |
| **Man. Turb., *H. d.*** | 0.69 | 0.1 | 42875 | 999 | 0 |
| **Mixed, MPB** | 0.30 | 0.6 | 42875 | 999 | 5 |
| **Mixed, *H. d.*** | -0.01 | 55.7 | 42875 | 999 | 556 |
| **MPB, *H. d.*** | 0.54 | 0.1 | 42875 | 999 | 0 |
