# Supplementary material for: Ecosystem engineers drive differing microbial community composition in intertidal estuarine sediments
Source: PLoS One. 2021 Feb 19;16(2):e0240952. doi: 10.1371/journal.pone.0240952 (PMC7895378; doi:10.1371/journal.pone.0240952)
Supplement: Attachment — Submitted filename: Response to reviewers.docx [file pone.0240952.s013.docx]

Response to reviewers (2)

**Reviewer #2: the paper has improved and comments have been addressed. I appreciate that niche concept has been diminished of importance. In my opinion I think that sticking witht the concept of ecosystem engineer is enough and clear. I have only one comment that concerns the aims and the experiment. Indeed, microphytobenthos is considered an explanatory variable and also a response variable. Could this point be adressed a bit better?**

This is a well-raised issue, that it is currently framed as both an explanatory and response variable.

In order to clarify this, the sentence:

Line 93: ‘Finally, by not adding macrofauna to mesocosms, the effect of microphytobenthos (MPB), were also studied.’

Has been changed to:

Finally, by not adding macrofauna to mesocosms, the effect of an uninterrupted microphytobenthos (MPB) biofilm, was also studied.

This highlights the fact that the MPB biofilms are present in the other treatments, and it is the lack of macrofauna that is the treatment, and the study of the MPB biofilm as a response variable is valid.

Also, at line 126: ‘Treatment 4 had no infauna added, and no physical turbation to allow MPB biofilms to develop ungrazed by infauna as with treatments 1-3, and undisturbed as with treatment 5, and is hereon referred to as the ‘MPB’ treatment.’ further highlights this point, and notes explicitly ‘MPB’ as an explanatory variable is again an absence of infauna, and is labelled as MPB for ease of understanding.
